# Predictive factors for successful weaning from mechanical ventilation in the internal medicine department

**DOI:** 10.1007/s11739-025-03860-3

**Published:** 2025-02-05

**Authors:** Gal Cohen, Idan Bergman, Alaa Atamna, Avishay Elis

**Affiliations:** 1 Department of Internal Medicine “C”, Rabin Medical Center, Beilinson Hospital, 49100 Petah Tikva, Israel; 2 Department of Infectious Diseases, Rabin Medical Center, Beilinson Hospital, Petah Tikva, Israel; 3 Faculty of Medical and Health Sciences, Tel Aviv University, Tel Aviv, Israel

**Keywords:** Mechanical ventilation, Weaning, Internal medicine departments, Predictive factors

## Abstract

The aging of the Israeli population along with a shortage of ICU beds have led to hospitalization of invasive mechanical ventilation patients in internal medicine departments, where, as opposed to ICU, the treatment is less than optimal. The aims of the study were to evaluate the predictive factors for successful weaning from mechanical ventilation in ventilated patients admitted to internal medicine departments. A retrospective study that included non-COVID 19 ventilated patients in internal medicine departments in a university affiliated hospital in Israel between the years 2018–2019. We compared datapoints between patients who were weaned from ventilators versus those who remained ventilated during the hospitalization, and defined demographic and clinical predictive factors for successful weaning. Data were collected from electronic medical records and included demographic, clinical, laboratory and ventilator information. The study group included 348 patients. The rate of successful weaning was 19%; patients who were successfully weaned were primarily functionally independent prior to ventilation, ventilated with low PEEP values, had high hemoglobin and albumin levels alongside with low CRP and lactate levels. Those who remained ventilated either required vasopressor treatment, had positive blood cultures or had lower GFR levels. The overall in-hospital mortality rate was 60%, while the 30-day mortality rate was lower in the extubated group [214 (76%) vs. 6 (9%), *P* < 0.0001]. Our findings highlight the low rate of weaning from ventilation in the department of medicine, with higher mortality rate among the remained ventilated patients. Various favorable clinical parameters might predict successful weaning.

## Introduction

The aging of the Israeli population along with a shortage of intensive care units (ICU) beds have led to hospitalization and invasive mechanical ventilation of critical care patients in internal medicine departments rather than in the ICU.

As opposed to the ICU, the treatment of critical care patients in the internal medicine department is less than optimal. The staff, including specialists, residents and nurses, are trained in internal medicine rather than intensive care; the nurse-to-patient ratio can reach 1:4–6; the on-call resident is responsible for a disproportional number of patients, reaching up to 40, including new admissions. In addition, there is a lack of advanced monitoring facilities, such as arterial lines and capnographs [1]. Consequently, younger healthier patients with a better prognosis are often treated in ICUs, while older fragile patients including those with cognitive and functional decline are admitted to the internal medicine wards despite their critical medical status [2].

Weaning from the ventilator is an essential aspect in the treatment of ventilated patients. Patients who are weaned demonstrate lower expected morbidity and mortality rates as well as less need for medical resources [3, 4].

The accepted criteria for weaning from mechanical ventilation include: an improvement in the initial reason for ventilation; ventilation parameters of: PEEP ≤ 5–8 cmH2O, FiO_2_40–50 ≥ % and pH7.25 ≤ ; hemodynamic stability, and the ability to breath spontaneously [5].

Naturally, it is more difficult to wean older patients who also possess a complex medical history and cognitive impairment, which can limit patient cooperation such as coughing on request [6, 7]. Moreover, elderly patients suffer from muscle atrophy, including of the respiratory muscles and diaphragm, as well as a decline in chest compliance [8]. Nevertheless, mental state deterioration is not considered a contraindication for weaning from ventilators [9].

The aims of the study were to assess the success rate of weaning ventilated patients from mechanical ventilation in the internal medical wards, to characterize those who were successfully weaned, and to predict the factors for successful weaning in the internal medicine department.

## Methods

A retrospective study of patients hospitalized in six internal medical wards at a university affiliated hospital, Rabin Medical Center, Beilinson Campus, Israel between January 1, 2018 and December 31, 2019.

The inclusion criteria included: age ≥ 18 years; ventilated outside the hospital, in the emergency department or in the internal medicine ward during the first three days of hospitalization; the reason for invasive ventilation was a respiratory failure due to acute and potentially reversible cause at the time of intubation [according our retrospective revision]. The exclusion criteria included: COVID 19 infection; admission to ICU while ventilated (primarily or after re ventilation) any time during hospitalization; mortality during the first 8 h of hospitalization.

Data were collected from the hospital electronic medical records, and included: demographic data: age, gender, functional status and place of residency prior to admission; BMI, Norton scale; past medical history (based on previous diagnosis), including: hypertension, diabetes mellitus (DM), ischemic heart disease (IHD), congestive heart failure (CHF), chronic renal failure (CRF), previous stroke, chronic obstructive pulmonary disease (COPD), malignancy (active/non active), organ recipient, liver cirrhosis, past mechanical ventilation; parameters and events during hospitalization, including: fever (maximum), use of vasopressors or dialysis, gastrointestinal bleeding (defined as using 80 mg IV Pantoprazole), bronchoscopy; ventilation parameters (maximum/average), including: ventilation volume and pressure, FiO2, PEEP; laboratory data (first and maximal/minimal, respectively) which included hemoglobin, creatinine, CRP, lactate and albumin levels; blood and sputum cultures results; length of hospitalization; success of weaning from ventilation; in-hospital and 30-days mortality.

The primary outcome was defined as successful weaning from ventilation during hospitalization. Secondary outcome included in-hospital and 30-days mortality rates.

Successful weaning was defined as weaning from invasive ventilation without the need for invasive re-ventilation.

### Statistical analysis

The statistical analysis was generated using R software, version 4.1.1 (The R Foundation) and R-studio version 2021.09.01, wording 372 (R Studio, Inc). Continuous data were expressed as median and range and were compared using Mann–Whitney *U* test. Chi-square test was used for comparing dichotomous variables.

In order to define the characteristics of successfully weaned patients and the predictors of such, those successfully weaned were compared to those who were not. A predictive model was built using logistic regression. The included variables in the multivariate backward stepwise model were those with statistically significant (*p* < 0.05) or with trend (*p* < 0.1) difference between the groups.

## Results

The study cohort included 348 patients, 66 (19%) of whom were successfully weaned from ventilation and 282 (81%) who were not.

The demographic and clinical characteristics of the entire cohort as well as between the successfully and unsuccessfully weaned groups are provided in Table 1. Half of the cohort were females and the mean age was 79 years (range 69–85). Before admission, two thirds lived at home, but less than a fifth (18.5%) were fully independent with a mean Norton scale of 9 (range 7–13). Around 14% of the cohort had been previously ventilated. When comparing the two groups, based on weaning success, the only parameter that significantly differed was the rate of full independent state prior to admission (28.8% vs. 16.1%, *p* = 0.027).

**Table 1:** Demographic and clinical data of all study group as well as based on successful weaning

	Total (*n* = 348)	Unsuccessful weaning (*n* = 282)	Successful weaning (*n* = 66)	*p* value
Age (years)	79.0 (69.085.0)	79.0 (69.0–86.0)	74.5 (70.2–83.8)	0.201
Female sex	176 (50.6%)	142 (50.4%)	34 (51.5%)	0.974
Independent in activities of daily living	64 (18.5%)	45 (16.1%)	19 (28.8%)	0.027
Arrival from				0.323
Home	232 (67.6%)	191 (68.7%)	41 (63.1%)	
Assisted living center	74 (21.6%)	60 (21.6%)	14 (21.5%)	
Other hospital	27 (7.9%)	21 (7.6%)	6 (9.2%)	
Other	10 (2.9%)	6 (2.2%)	4 (6.2%)	
BMI (kg/cm^2^)	26.0 (22.8–30.1)	26.0 (22.8–31.1)	25.7 (23.9–29.1)	0.621
Norton value	9.0 (7.0–13.0)	9.0 (7.0–13.0)	9.0 (7.0–11.0)	0.394
Norton value < 14	281 (81.9%)	224 (80.6%)	57 (87.7%)	0.245
Past medical history				
Hypertension	124 (35.6%)	100 (35.5%)	24 (36.4%)	1.000
Diabetes mellitus	74 (21.3%)	63 (22.3%)	11 (16.7%)	0.397
Coronary heart disease	42 (12.1%)	34 (12.1%)	8 (12.1%)	1.000
Heart failure	32 (9.2%)	25 (8.9%)	7 (10.6%)	0.838
Chronic kidney disease	31 (8.9%)	27 (9.6%)	4 (6.1%)	0.508
Cerebrovascular accident	30 (8.6%)	27 (9.6%)	3 (4.5%)	0.286
Chronic obstructive pulmonary disease	29 (8.3%)	23 (8.2%)	6 (9.1%)	1.000
Non-active malignancy	25 (7.2%)	22 (7.8%)	3 (4.5%)	0.511
Organ transplant	13 (3.7%)	13 (4.6%)	0 (0.0%)	0.156
Asthma	10 (2.9%)	9 (3.2%)	1 (1.5%)	0.746
Liver cirrhosis	4 (1.1%)	4 (1.4%)	0 (0.0%)	0.740
Past mechanical ventilation	51 (14.7%)	42 (14.9%)	9 (13.6%)	0.947

Table 2 describes the key events during hospitalization. Around half of the patients were treated using vasopressor agents, around 10% had GI bleeding and 15% had positive blood cultures. Prominent differences between patients who were weaned from ventilation and those who were not included: use of vasopressors (18.2% vs. 52.1%, *p* < 0.001, respectively) and positive blood cultures (6.1% vs. 16.3%, *p* = 0.052, respectively). Length of hospitalization was significantly longer [10.0 (7.0–18.8) vs. 7.0 (3.0–15.0) days, *p* < 0.001, respectively].

**Table 2:** Events during ventilation

	Total (*n* = 348)	Unsuccessful weaning (*n* = 282)	Successful weaning (*n* = 66)	*p* value
Temperature (C°)	37.4 (37.0–38.0)	37.4 (37.0–37.9)	37.4 (37.1–38.0)	0.655
Use of vasopressors	159 (45.7%)	147 (52.1%)	12 (18.2%)	< 0.001
Hemodialysis	15 (4.3%)	13 (4.6%)	2 (3.0%)	0.816
Gasrointestinal bleeding*	34 (9.8%)	28 (9.9%)	6 (9.1%)	1.000
Bronchoscopy	21 (6.0%)	19 (6.7%)	2 (3.0%)	0.395
Positive blood Cultures	50 (14.4%)	46 (16.3%)	4 (6.1%)	0.052
Positive sputum Cultures	131 (37.6%)	111 (39.4%)	20 (30.3%)	0.220
Duration of ventilation (days)	2.0 (0.0–6.0)	2.0 (0.0–8.0)	2.0 (0.2–3.0)	0.153
Length of stay (days)	8.0 (3.0–16.0)	7.0 (3.0–15.0)	10.0 (7.0–18.8)	< 0.001

*Gastrointestinal bleeding—according to administration of 80 mg Pantoprazole IV

Table 3 describes the ventilation measures and a comparison between the study groups. No major differences were found between the groups with regards to ventilation modes. The only parameter that was statistically, but not clinically, different was the maximal PEEP—which was lower in the weaned group [5.0 (5.0–5.0) vs. 5.0 (5.0–6.8) cmH2O, *p* = 0.004].

**Table 3:** Ventilation settings

	Overall (*n* = 348)	Unsuccessful weaning (*n* = 282)	Successful weaning (*n* = 66)	*p*
Volume, maximum (ml)	535.0 (471.0–644.0)	543.0 (468.0–649.0)	518.5 (472.2–615.0)	0.550
Volume, last Before weaning (ml)	467.0 (402.0–523.0)	465.0 (400.8–523.8)	472.0 (403.0–522.0)	0.786
Inspiratory pressure, maximal (cmH2O)	22.0 (18.0–26.0)	22.0 (18.0–27.0)	21.0 (17.5–24.0)	0.485
Inspiratory pressure, last before weaning (cmH2O)	19.8 (4.6%)	20.2 (4.3%)	19.4 (5.0%)	0.419
Pressure support, maximal (cmH2O)	15.0 (10.0–18.0)	15.0 (10.0–18.8)	14.5 (12.0–17.0)	0.294
Pressure support, last before weaning (cmH2O)	13.5 (12.0–15.0)	14.0 (10.0–16.0)	13.0 (12.0–15.0)	0.857
FiO2, last before weaning (%)	40.0 (31.5–45.0)	40.0 (31.0–44.0)	40.0 (35.0–50.0)	0.218
PEEP, maximal (cmH2O)	5.0 (5.0–6.0)	5.0 (5.0–6.8)	5.0 (5.0–5.0)	0.004
PEEP, last before weaning (cmH2O)	5.0 (5.0–5.0)	5.0 (5.0–5.0)	5.0 (5.0–5.0)	0.994

Several laboratory parameters were associated, not always with clinical meaning, with successful weaning from ventilation, including: greater hemoglobin level at admission and greater ones during hospitalization [12.5 g/dL (10.9–13.8) vs. 11.8 g/dL (10.1–13.3), *p* = 0.04 and 10.0 g/dL (8.4–11.3) vs. 9.1 g/dL (7.6–11.0), *p* = 0.08, respectively]; lower CRP levels at admission and lower peak values during hospitalization [2.2 mg/dL (0.8–6.2) vs. 4.2 mg/dL (1.4–11.4), *p* = 0.008 and 12.8 mg/dL (4.4–23.3) vs. 16.8 (7.4–27.6), *p* = 0.076, respectively]; lower maximal lactate levels during hospitalization [29.0 mg/dL (24.0–35.0) vs. 34 mg/dL (25.0–60.0), *p* = 0.005]; higher albumin levels at admission and during hospitalization [3.9 g/dL (3.5–4.3) vs. 3.6 g/dL (3.1–4.0), *p* < 0.0001 and 3.1 g/dL (2.8–3.4) vs. 2.5(2.1–3.0), *p* < 0.0001, respectively]. Lower minimal GFR levels during hospitalization were associated with lack of weaning [54.2 ml/min (38.9–70.7) vs. 31.2 ml/min (19.9–61.2), *p* = 0.007].

**Fig. 1:**
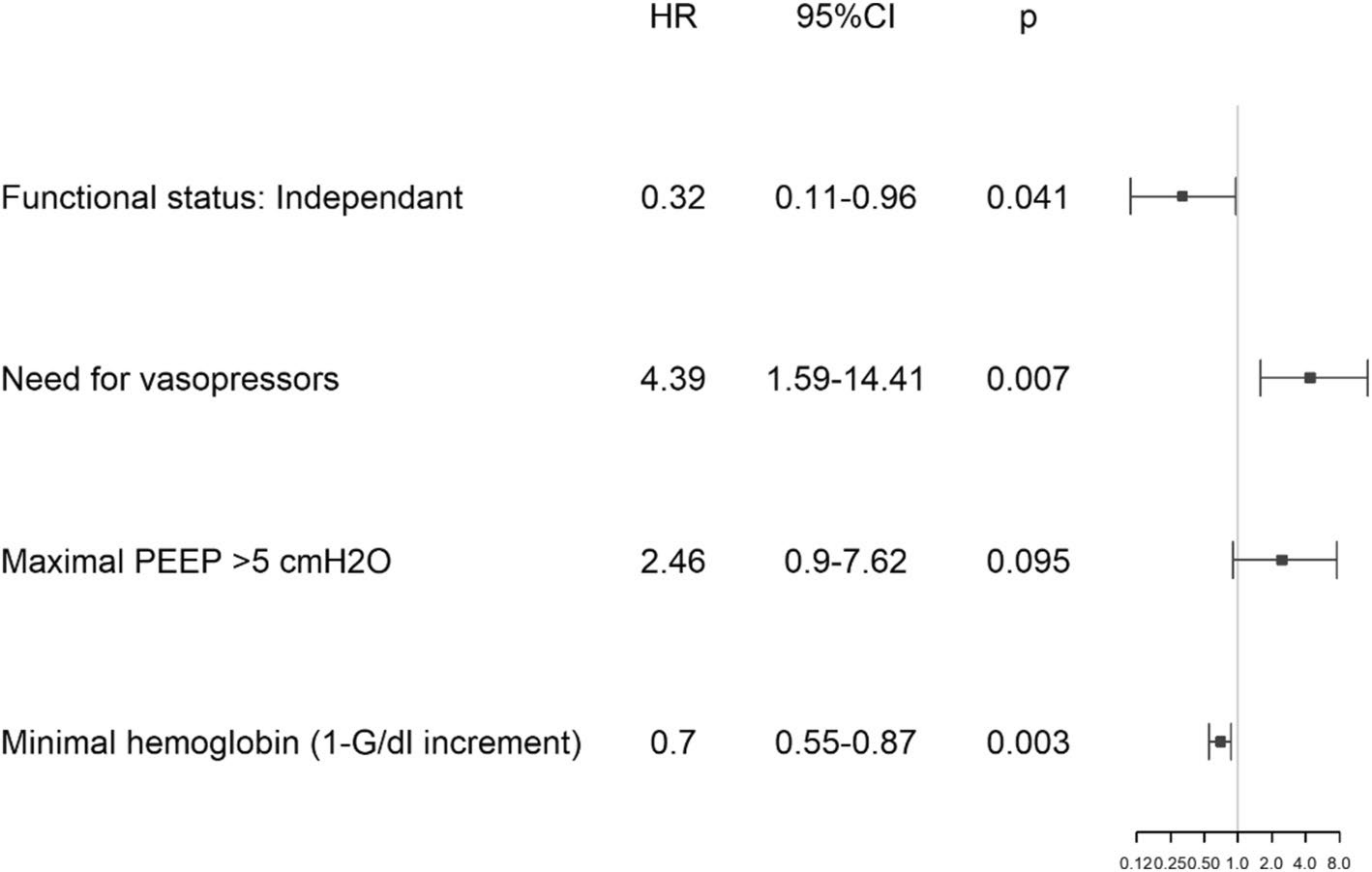
Factors that significantly predict successful weaning from ventilation. *PEEP* Positive end-expiratory pressure

Figure 1 demonstrates the factors that significantly predicted weaning from ventilation. Independent functional status prior to hospitalization significantly predicted weaning [HR 0.32 (0.11–0.96], while the use of vasopressor agents predicted failure [HR 4.39 (1.59–14.41)].

Amongst the entire cohort (*n* = 348), 81% were not weaned from ventilation, 9.5% were re-ventilated, 60% died during hospitalization, and 39% of the 138 discharged patients were ventilated upon discharge, all through tracheostomy (data not shown).

Most of the in-hospital deaths (*n* = 82, 83%) occurred during the first 15 days of hospitalization.

At 30 days, 214 (76%) of those who remained ventilated after discharge died, versus only 6 (9%) of those who were weaned prior to discharge.

## Discussion

This study evaluated patients who were ventilated in internal medicine wards.

The main results included: a 19% rate of successful weaning from ventilation; weaned patients were mostly fully independent prior to ventilation, ventilated with low PEEP values, had high hemoglobin and albumin levels alongside low CRP and lactate levels. Moreover, those who remained ventilated tended the need of vasopressor treatment, had positive blood cultures and lower GFR levels during hospitalization. Finally, in-hospital mortality was 60%, while the 30-days mortality rate was lower in the ones who were weaned from ventilation.

As previously hypothesized, the study cohort of ventilated patients in internal medicine wards are older, with a mean age of 79 years (range 69–85) and a low rate of independence prior to admission. Lieberman et al., found that the decision to ventilate in ICUs was significantly and independently influenced by age and a pre-hospitalization functional independence measure. In that study, the percentage of ICU ventilations in the 65 to 74, 75 to 84, and 85 + age groups was 62%, 45%, and 23%, respectively [2]. We assume that as a consequence, t older patients are treated and ventilated at the departments of medicine.

About one fifth of the patients in our study were successfully weaned from the ventilator. A similar finding was described by Xiao K et al. concerning adults (age > 60) with multiple organ dysfunction syndrome [10].

Esteban et al. concluded that survival among mechanically ventilated patients depends not only on the factors present at the start of mechanical ventilation (such as prior functional status characterized by limited activity), but also on the development of complications and patient management in the ICU, like the use of vasoactive drugs [3]. Fujii M et al. described that in ventilated adults over the age of 65 with community-acquired pneumonia, a low concentration of serum albumin, which is a marker for nutritional and inflammatory status, was associated with significant difficulty in weaning from mechanical ventilation [11]. Our study results support these findings and add several more parameters that might predict weaning from ventilation, such as higher hemoglobin and lower lactate and CRP levels. Although we found that the trough hemoglobin level during admission is a predicting factor to the ability to wean from mechanical ventilation (for every 1 g/dl increment of minimal hemoglobin level the risk of failure in weaning is reduced by 30%), low hemoglobin levels are not an absolute contraindication for weaning [5].

Another important finding was the trend towards failure in weaning in cases of high PEEP values ventilation, especially above 5 cmH2O. This might represent a severe oxygenation disorder in these patients which may pose a challenge to withdraw from mechanical ventilation.

Nearly half of our study population were hemodynamically unstable and needed vasopressors support. Despite this, the main vasopressor in use was dopamine, which is not the first-line vasopressor therapy considered for septic shock [12].

The relationship between pH and arterial blood gas tests with our study outcomes were not included in the study because of their relatively limited use in internal medicine wards and preference for venous blood sampling.

Hersch et al. reported that in-hospital survival rates (discharged alive from hospital) among ventilated patients in the internal medicine wards was 20% compared to 38% in ICU-ventilated patients [1]. In comparison, Lieberman et al. demonstrated an in-hospital mortality rate of 53.0% in ICUs compared with 68.2% in non-ICU wards, however, these results were not independently and significantly affected by hospitalization in ICUs [2]. Our study results demonstrate similar results of an approximate 60% in-hospital mortality rate amongst internal medicine patients.

Another finding of our study revealed that the length of hospitalization among patients weaned from ventilation was significantly longer than those who remained ventilated. This finding can be explained by the time necessary for recovery from ventilation due to de-conditioning in the weaned group.

This study is one of only few to have surveyed ventilated patients in internal medicine wards. This reality largely exists only in Israel and was considered one of the advantages of the Israeli medical system during the COVID-19 pandemic. Importantly, the need to ventilate patients in internal medicine departments might exist during future pandemics or in pressing situations, hence the importance of this study and its results.

There are several limitations to our study. Firstly, its retrospective design and its limited randomized selection and confounders. Second, the exact causes of admission and ventilation were not detailed, mainly because of the retrospective design and overlap causes, like: pneumonia, sepsis, acute CHF and COPD exacerbation. Furthermore, the weaning process from ventilation was not preformed according to a structured protocol and was mostly based on the clinical judgment of the treating physicians, this fact might affect the study's outcomes. Future prospective research should include a structured weaning protocol. Lastly, the use of un accepted definition of gastrointestinal bleeding (using 80 mg IV Pantoprazole) mainly because of the retrospective design and lack of data.

In conclusion, the rate of weaning from ventilation in the department of medicine is low, with higher mortality rates. Various favorable clinical parameters might predict successful weaning.
